# IgLON5 deficiency produces behavioral alterations in a knockout mouse model

**DOI:** 10.3389/fimmu.2024.1347948

**Published:** 2024-02-02

**Authors:** Jon Landa, Ana Beatriz Serafim, Mercedes Alba, Estibaliz Maudes, Laura Molina-Porcel, Anna Garcia-Serra, Francesco Mannara, Josep Dalmau, Francesc Graus, Lidia Sabater

**Affiliations:** ^1^ Neuroimmunology Program, Fundació de Recerca Clínic Barcelona-Institut d'Investigacions Biomédiques August Pi i Sunyer-Caixa Research Institute (CRI), Universitat de Barcelona, Barcelona, Spain; ^2^ Alzheimer’s Disease and Other Cognitive Disorders Unit, Neurology Service, Hospital Clínic, Institut d'Investigacions Biomèdiques August Pi i Sunyer (IDIBAPS), Barcelona, Spain; ^3^ Neurological Tissue Bank, Biobanc, Hospital Clínic, Institut d'Investigacions Biomèdiques August Pi i Sunyer (IDIBAPS), Barcelona, Spain; ^4^ Department of Neurology, University of Pennsylvania, Philadelphia, PA, United States; ^5^ Centro de Investigación Biomédica en Red, Enfermedades Raras (CIBERER), Madrid, Spain; ^6^ Catalan Institution for Research and Advanced Studies (ICREA), Barcelona, Spain

**Keywords:** Iglon5, animal model, knockout, behavior (mice), anti-IgLON5 antibody encephalopathy

## Abstract

**Background:**

Anti-IgLON5 disease is a neurological disorder characterized by autoantibodies against IgLON5 and pathological evidence of neurodegeneration. IgLON5 is a cell adhesion molecule of unknown function that is highly expressed in the brain. Our aim was to investigate the impact of IgLON5 loss-of-function in evaluating brain morphology, social behavior, and the development of symptoms observed in an IgLON5 knockout (IgLON5-KO) mouse model.

**Methods:**

The IgLON5-KO mice were generated using CRISPR-Cas9 technology. Immunohistochemistry on fixed sagittal brain sections and Western blotting brain lysates were used to confirm IgLON5 silencing and to evaluate the presence of other cell surface proteins. Two- month-old IgLON5-KO and wild-type (WT) mice underwent a comprehensive battery of behavioral tests to assess 1) locomotion, 2) memory, 3) anxiety, 4) social interaction, and 5) depressive-like behavior. Brain sections were examined for the presence of anatomical abnormalities and deposits of hyperphosphorylated tau in young adult (2-month-old) and aged (22-month-old) mice.

**Results:**

Mice did not develop neurological symptoms reminiscent of those seen in patients with anti-IgLON5 disease. Behavioral testing revealed that 2-month-old IgLON5-KO mice showed subtle alterations in motor coordination and balance. IgLON5-KO females exhibited hyperactivity during night and day. Males were observed to have depressive-like behavior and excessive nest-building behavior. Neuropathological studies did not reveal brain morphological alterations or hyperphosphorylated tau deposits.

**Conclusion:**

IgLON5-KO mice showed subtle alterations in behavior and deficits in fine motor coordination but did not develop the clinical phenotype of anti-IgLON5 disease.

## 1 Introduction

Anti-IgLON5 disease is a neurological disorder characterized by the presence in serum, and >90% of cases are in cerebrospinal fluid (CSF), with IgG antibodies targeting IgLON5, a neuronal cell adhesion molecule of unknown function. The clinical manifestations of anti-IgLON5 disease are heterogeneous, and most patients present a combination of symptoms that include a sleep disorder with non-rapid eye movement (REM) and REM parasomnias, stridor or obstructive sleep apnea, gait instability, abnormal movements, bulbar symptoms, and cognitive impairment (1–3). Motor neuron disease-like phenotype, mimicking amyotrophic lateral sclerosis, has been also recently described in some patients with anti-IgLON5 disease (4, 5).

In at least 75% of patients, symptoms progress slowly for months or years, a feature that is unusual in other autoimmune encephalitis with antibodies against surface antigens, which often develop in a matter of a few weeks (6). Initial neuropathological findings revealed features of neurodegeneration with deposits of hyperphosphorylated tau in the neurons of the hypothalamus and tegmentum of the brainstem with a rostro-caudal gradient of severity (1, 7). Although the pathogenesis of anti-IgLON5 disease remains unclear, there is strong evidence that it has an autoimmune origin: 1) anti-IgLON5 disease has a robust genetic association with HLA-DRB1*10:01-DQB1*05:01 haplotype (8).; 2) some autopsy studies have not shown abnormal deposits of hyperphosphorylated tau in all patients, suggesting that this is a late event in the disease and not the primary cause (9, 10); 3) *in vitro* experiments of cultured rat brain neurons demonstrated that IgLON5 antibodies produce an irreversible reduction of membrane IgLON5 clusters (11), in addition to cytoskeletal lesions, such as dystrophic neurites and axonal swellings (12).

IgLON5 is the fifth member of the IgLON family, which belongs to the immunoglobulin superfamily of cell adhesion molecules. IgLONs are involved in several physiological processes, including cell adhesion, migration, and neurite outgrowth (13–17). In addition to IgLON5, other members of the IgLON family include the opioid-binding cell adhesion molecule (OBCAM/IgLON1), neurotrimin (NTM/IgLON2), limbic system-associated member protein (LSAMP/IgLON3), and neural growth regulator 1 (NEGR1/IgLON4). IgLONs have three immunoglobulin-like domains in the extracellular region (ectodomain), which are attached to the membrane by a glycosylphosphatidylinositol (GPI) anchor. IgLON5, like the other IgLON family members, is secreted by ectodomain shedding, an important post-translational mechanism involved in neuronal plasticity, axonal guidance, and cell migration. IgLON5 predominantly establishes homomeric and heteromeric *cis-* (within the cell) and *trans-* (between cells) interactions with other IgLON family members that can be disturbed by IgLON5 antibodies (18–20).

Despite the *in vitro* effects of IgLON5 antibodies, passive transfer experiments using cerebroventricular infusion or intracerebral injection of IgLON5-IgG have failed to reproduce the most characteristic symptoms of the disease and have been unable to demonstrate *in vivo* the pathogenic effects described *in vitro* (21, 22).

The limited knowledge of the physiological function of IgLON5 represents a major drawback to studying the pathogenic mechanisms of IgLON5 antibodies and the relation with the tauopathy observed in some patients. Knockout (KO) mice are a powerful tool for understanding the physiologic mechanisms of the suppressed protein and the symptoms derived from its loss. The aim of this study was to investigate the impact of IgLON5 loss-of-function on brain morphology, social behavior, and the development of clinical symptoms observed in anti-IgLON5 disease in an IgLON5-KO mouse model.

## 2 Materials and methods

### 2.1 Animals

IgLON5 KO mice C57BL/6NCrl-Iglon5em1(IMPC)Mbp/Mmucd (IgLON5-KO) were generated using CRISPR-Cas9 system at the Mutant Mouse Resource and Research Center (MMRRC; https://www.mmrrc.org/catalog/sds.php?mmrrc_id=50638) in the University of California, Davis (UCD). Exon 3 was deleted using two guide RNA sequences (CTGGAAGCTAGACTTCTGAGGGG and GTGCCCTGCTGAATACCATAAGG) and Cas9 nuclease, introducing a frameshift and creating a premature stop codon. A colony of IgLON5-KO and heterozygous IgLON5−/+ (IgLON5-HET) mice was established locally from the founders. Wild-type (WT) mice with the same genetic background (C57BL/6N) were used as controls and for crossing back with IgLON5-KO to breed IgLON5-HET. Aged IgLON5-KO animals (22-month-old) were used to determine the presence of neurodegenerative features. A maximum of five animals were housed per cage. The room was maintained at controlled temperature (21°C) and humidity (55% ± 10%) with illumination at 12-h cycles; food and water were available *ad libitum*. All procedures were conducted in accordance with standard ethical guidelines and approved by the local ethics committee of the University of Barcelona (procedure code: 10903).

### 2.2 Behavioral studies

Behavioral testing was performed in 8-week-old female and male mice (25–30 g; KO, n = 27 (16 male and 11 female); WT, n = 24 animals (10 male and 14 female)) to study the following: 1) locomotion (beam balance test, gait analysis, and locomotor activity in activity boxes during 24 hours), 2) memory (novel object location), 3) anxiety (black and white and open field test), 4) social interaction (social interaction test and nest building), and 5) depressive-like behavior (anhedonia and tail suspension test). Prior to the testing period, all mice were handled and acclimated to the experimental room for 1 week. Animals’ weight was registered every week during the behavioral evaluation. Non-significant behavioral results are summarized in **Table 1**. IgLON5-HET animals (n = 20 animals) had no differences from WT animals in behavioral studies (data not shown).

**Table 1:** Non-significant behavioral test results.

Parameter	WT male (n = 10)	WT female (n = 14)	KO male (n = 16)	KO female (n = 11)	Statistics (genotype)
Weight (g)	27.43 ± 0.502	23.57 ± 0.312	29.19 ± 0.679	24.04 ± 0.704	p = 0.1103
Brain weight (g)	0.468 ± 0.017	0.471 ± 0.016	0.477 ± 0.015	0.477 ± 0.020	p = 0.1524
Novel object location (NOL)					
Exploration time (s)	32.03 ± 13.081	26.3 ± 15.477	23.14 ± 11.514	16.74 ± 7.018	p = 0.0629
NOL index	0.100 ± 0.155	0.240 ± 0.090	0.214 ± 0.113	0.167 ± 0.168	p = 0.6698
Gait analysis					
Right forelimb stride (cm)	8.24 ± 1.384	7.566 ± 0.706	8.169 ± 0.779	7.941 ± 0.495	p = 0.5470
Left forelimb stride (cm)	8.293 ± 1.314	7.564 ± 0.646	8.298 ± 0.879	7.921 ± 0.509	p = 0.4731
Right hind limb stride (cm)	8.118 ± 1.373	7.541 ± 0.671	8.041 ± 0.798	7.868 ± 0.58	p = 0.6223
Left hind limb stride (cm)	8.161± 1.287	7.576 ± 0.491	8.029 ± 0.897	7.689 ± 0.762	p = 0.9706
Forelimb stride width (cm)	1.433 ± 0.116	1.422 ± 0.141	1.435 ± 0.105	1.363 ± 0.09	p = 0.3935
Hind limb stride width (cm)	2.729 ± 0.21	2.623 ± 0.243	2.755 ± 0.297	2.578 ± 0.197	p = 0.8986
Black and white					
Entries in white	8.333 ± 4	11.846 ± 3.436	7.933 ± 4.511	11.714 ± 4.151	p = 0.8363
Entries in distal white	5.777 ± 3.032	9.076 ± 3.546	8.066 ± 3.411	8.571 ± 3.552	p = 0.4108
Time in white (%)	25.966 ± 11.861	30.421 ± 12.765	33.998 ± 15.833	30.396 ± 7.074	p = 0.3391
Time in distal white (%)	7.995 ± 3.828	9.921 ± 6.466	11.4 ± 6.082	9.791 ± 5.094	p = 0.3686
Open field					
Entries to medial	20.1 ± 7.622	20.9 ± 10.514	23.812 ± 9.224	21.909 ± 7.674	p = 0.3756
Entries to central	4.6 ± 2.319	5.7 ± 4.347	5.312 ± 2.725	5.454 ± 3.615	p = 0.8114
Time out of peripheral (%)	12.767 ± 9.1	10.034 ± 7.477	15.896 ± 6.31	9.68 ± 3.965	p = 0.4976
Social interaction test					
Trial 1 (s)	46.028 ± 9.916	48.417 ± 10.152	46.645 ± 9.545	43.688 ± 12.619	p = 0.3317
Trial 2 (s)	43.546 ± 13.321	45.811 ± 13.859	38.921 ± 15.671	38.43 ± 14.924	
Trial 3 (s)	29.22 ± 18.08	41.332 ± 17.532	34.265 ± 17.312	31.095 ± 15.497	
Trial 4 (s)	30.177 ± 27.804	35.679 ± 18.793	44.206 ± 11.141	29.068 ± 18.199	
Trial 5 (s)	45.535 ± 11.828	41.38 ± 20.58	38.145 ± 25.539	37.151 ± 12.056	
Tail suspension test					
Immobility time (s)	60.34 ± 34.278	134.264 ± 41.748	66.118 ± 40.957	133.4 ± 32.639	p = 0.8230
Energy (unit)	31,889.79 ± 16,486.651	11,527.914 ± 6,410.089	34,514.793 ± 19,148.298	12,493.654 ± 6,083.17	p = 0.6483
Power (unit)	403.58 ± 231.086	203.107 ± 135.828	425.031 ± 221.444	173.436 ± 74.365	p = 0.9362

WT, wild type; KO, knockout.

#### 2.2.1 Novel object location

Animals were habituated to an empty, square arena (25 × 25 cm) with visual cues for 10 minutes twice daily for 4 days. On the day of the test, the animals were placed in the arena with two equal objects in two opposite corners, and they were allowed to explore the objects for 9 minutes (familiarization phase). After 3 hours, the animals were reintroduced to the arena, where one of the objects had been relocated to a different corner. The animal was given 9 minutes to explore the objects (test phase), and the time spent exploring each object was recorded. The following formula was used to compute a discrimination index (novel object location (NOL) index): time of exploration of the moved object minus the time of exploration of the not moved object, divided by the total time of exploration of both objects. A higher discrimination index indicates a better memory of the position of both objects.

#### 2.2.2 Gait assessment

Animals were trained to run through a 50 × 8 cm corridor with a box in the end. The procedure was repeated twice to habituate the mice to the environment and avoid anxiety. The following day, filter paper was placed on the corridor, and animal paws were painted with non-toxic paint to identify footstep positions. Distance traveled for each paw and distance between opposite paws were measured for each animal. A minimum of four consecutive strides per animal were recorded.

#### 2.2.3 Beam balance test

A 1-m beam with a flat surface of 12-mm width was placed on two poles resting 50 cm above the tabletop. On one pole, a lamp was used as an aversive stimulus (starting point), while food pellets in a black box were placed on the other pole to attract mice (endpoint). For habituation, animals were placed in the middle of the beam and left to cross half the beam. If animals performed the task correctly, they were placed further from the endpoint until they learned to cross the whole beam. Once they performed the task correctly a minimum of three times, the beam was substituted with a 6-mm-wide round beam, and animals performed the task twice. The next day, animals were submitted to the test and placed on the aversive pole, and the time to cross the beam and the number of slips were measured.

#### 2.2.4 Black and white test

The black and white test box was composed of two compartments (20 cm wide, 20 cm long, and 30 cm high) connected by a tunnel that was 6 cm wide and 6 cm high. One compartment was painted black and kept at 10 lux, while the other was painted white, brightly illuminated (500 lux), and subdivided into three sections based on distance from the tunnel (distal, medial, and proximal). Animals were initially placed in the black compartment, creating a conflict between the natural tendency of rodents to explore new environments and the tendency to avoid brightly illuminated areas. Anxiety levels were automatically measured using SMART software (Panlab, Harvard Apparatus, Barcelona, Spain), quantified as the percentage of time spent in the light box and the number of entries made into each section.

#### 2.2.5 Locomotor activity

Spontaneous locomotor activity was automatically analyzed using activity boxes (9 × 20 × 11 cm; Imetronic, Pessac, France) equipped with two rows of photocell detectors. Animals’ locomotion was evaluated over 24 hours, divided into 12 hours of light and 12 hours of darkness. Activity was quantified by counting the number of times the animal crossed the detector. Day and night cycles were analyzed separately.

#### 2.2.6 Open field test

Animals were placed in a brightly illuminated (500 lux) round arena of 25-cm diameter, divided into three concentric circles (peripheral, medial, and central). Mice were free to explore the arena for 5 minutes. Anxiety levels were determined based on the time spent in the peripheral zone, as well as the number of entries made into each section. Tracking of the mice was automatically measured using SMART software (Panlab, Harvard Apparatus).

#### 2.2.7 Sucrose preference test

Anhedonia was measured by the preference for sucrose. Two bottles containing water that were swapped positions daily were placed in the cage, one with 2% sucrose and one without. The consumption from each bottle was recorded over 5 days. On the fifth day, the mice’s sucrose preference was calculated as the ratio of water with sucrose consumed to the total liquid (water with and without sucrose) consumed. A lower ratio is indicative of depressive-like behavior (23).

#### 2.2.8 Social interaction test

Individualized mice (1-week minimum) were exposed to a mouse of the same sex and similar age for 1 minute and then removed from the interaction cage. The same intruder was introduced four times separated by intervals of 10 minutes. In the fifth trial, the intruder was changed to a different mouse. Trials were recorded, and the total time that the experimental mouse interacted with the intruder mouse was measured. Development of social memory was indicated by a reduction of exploration time with the same animal (trials 1–4) and increased exploration time during the interaction with a new animal.

#### 2.2.9 Nest-building test

A nest-building test was performed by introducing 9 g of Nestlets material (Ancare, Bellmore, NY, USA) overnight in the cage of individualized mice. The following day, the nest was scored using a previously described scale, and unused Nestlets material was weighted (24).

#### 2.2.10 Tail suspension test

Mice were suspended by adhesive tape applied to the tail in an automatic system for 6 minutes (BIO-TST, Bioseb, Vitrolles, France). The total periods of immobility, energy, and power were automatically recorded during the experimental period. Prolonged periods of immobility are indicators of helplessness, a characteristic of depressive-like behavior (25).

### 2.3 Brain tissue processing

Animals were deeply anesthetized with isoflurane and perfused with phosphate-buffered saline (PBS) after the last behavioral test. Brains were removed and weighed. For analysis of membrane proteins, brains were cut sagittally. One hemisphere was dissected to obtain protein extracts of the hippocampus, cerebellum, and brainstem; the other was processed for the immunohistochemistry analysis of cell surface proteins following previously described protocols (26). To investigate the presence of signs of neurodegeneration in aged animals, 22-month-old mice (IgLON5-KO, IgLON5-HET, and WT) underwent trans-cardiac perfusion with PBS fixed in 4% paraformaldehyde (PFA) for 12 hours, cut into 2-mm coronal sections, and incubated in increasing concentrations of alcohol (70% for 3 hours twice, 96% overnight, and 100% for 3 hours); after an additional 3 hours in xylene, tissue was embedded in paraffin and kept at room temperature until used.

### 2.4 Immunohistochemistry

Immunohistochemistry to detect neuronal cell-surface proteins was performed using a standard avidin–biotin–peroxidase method as previously described on 7-µm frozen sagittal rat brain sections post-fixed with PFA. The following commercial antibodies were used: 1:500 diluted IgLON5 (ab122763, abcam, Cambridge, UK), IgLON1 (STJ94590), IgLON2 (STJ94442), IgLON3 (STJ116461), IgLON4 (STJ94394, St. John’s Laboratory, London, UK), KIDINS220 (21856-1-AP, Proteintech, Rosemont, IL, USA), IGSF21 (21465-1-AP; Proteintech), and CACNA2D2 (SAB1401461, Sigma-Aldrich, Darmstadt, Germany); serum (1:200 diluted) or CSF (1:5 diluted) from patients containing IgLON5, NMDAR, GABABR, and GABAAR antibodies was used also to investigate their presence in IgLON5-KO brains.

To study the brain structure and presence of tau hyperphosphorylation in aged animals, 4-μm-thick coronal sections were deparaffinized in xylene, rehydrated in alcohol, washed in tap water, and heated for 2 min in a pressure cooking oven in 0.1 M sodium citrate buffer (pH 6.0). Sections were then stained with hematoxylin and eosin or processed for immunohistochemistry. Phospho-tau (ser202, Thr205) monoclonal antibodies (AT8, MN1020, Thermo Fisher Scientific, Waltham, MA, USA) or neurofilament medium staining (ab7794, abcam, 1:5,000 diluted) was incubated overnight at 4°C (1:1,000 diluted) followed by goat anti-mouse biotinylated as secondary antibody (1:1,000 diluted), revealed by a standard avidin–biotin–peroxidase method and visualization by diaminobenzidine. A section of the prefrontal cortex from an Alzheimer’s disease patient was included as a positive control of tau hyperphosphorylation and incubated with AT8 antibody as described above.

### 2.5 Western blotting

Dissected brain regions were homogenized in standard lysis buffer (Tris-HCl 50 mM, NaCl 150 mM, EDTA 5 mM, 1% triton X-100, and protease inhibitor cocktail 1:50 diluted) by sonication. Lysates were centrifuged at 16,100 *g* for 15 minutes, and the supernatant was kept. The concentration of protein was determined using a standard bicinchoninic acid (BCA) assay, and 10 µg of protein was loaded into a gel and, after electrophoresis, transferred to a nitrocellulose membrane (1704158, Bio-Rad, Hercules, CA, USA). Anti-IgLON5 (abcam), beta-actin (Sigma-Aldrich), IgLON5 (ab122763, abcam, Cambridge, UK), IgLON1 (STJ94590), IgLON2 (STJ94442), IgLON3 (STJ116461), and IgLON4 (STJ94394) were incubated in 1/1,000 dilution overnight at 4°C followed by the appropriate secondary antibody (anti-rabbit-horseradish peroxidase (HRP) or anti-mouse-HRP) and were revealed following a standard enhanced chemiluminescence developing kit (RPN2108, GE Healthcare, Chicago, IL, USA).

### 2.6 Statistical analysis

Statistical analyses were performed using GraphPad Prism 7. Results are expressed as mean values ± SD and outliers (**Table 1**). To analyze data from behavioral studies, a two-way ANOVA model with Sidak’s multiple comparison tests was performed including the between‐subjects factors experimental group and sex. If the test had multiple time points, a three-way ANOVA model with Tukey’s multiple comparisons was applied to observe only robust differences.

## 3 Results

### 3.1 IgLON5-KO mouse phenotype description and histological studies

**Figure 1:**
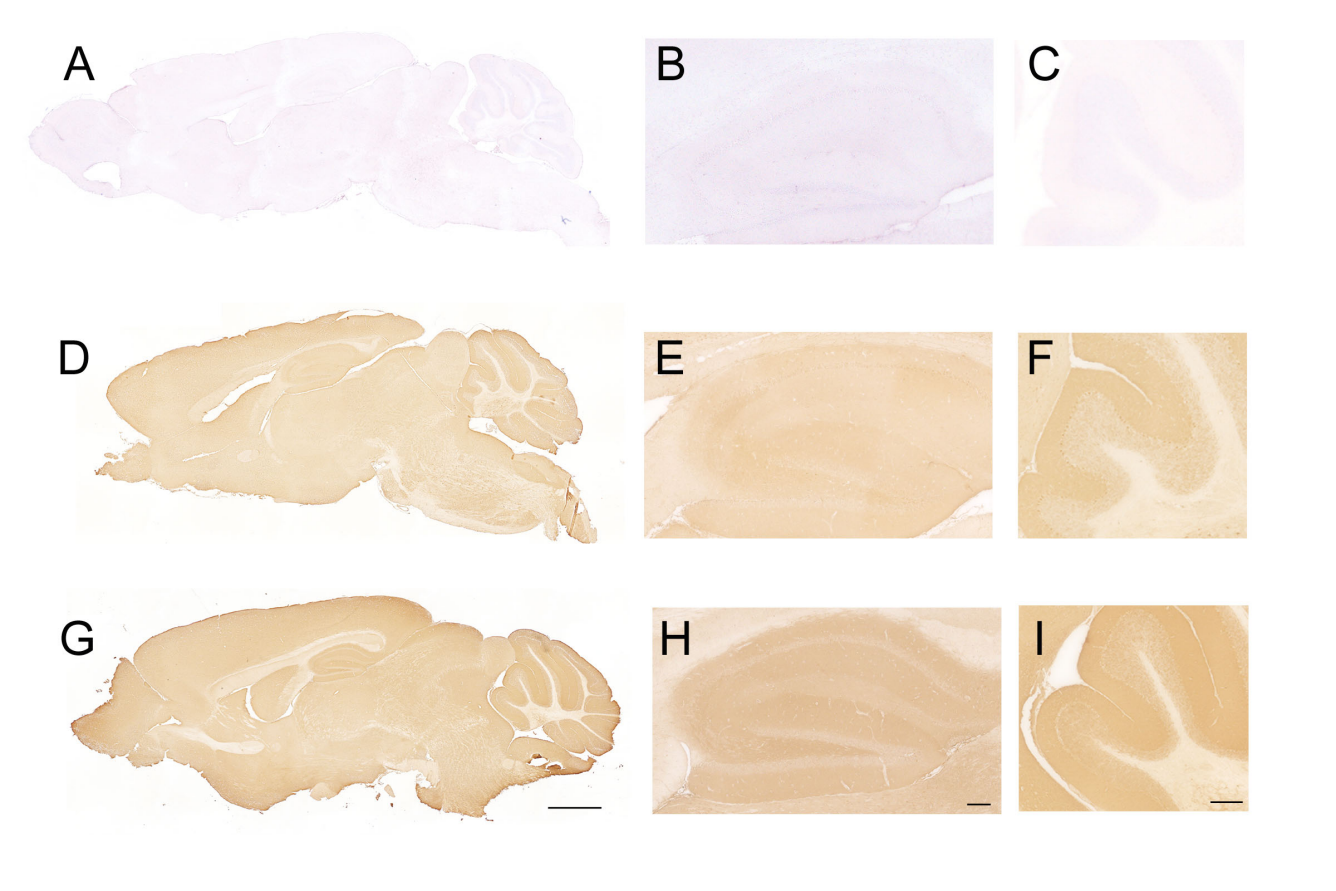
Immunohistochemistry on sagittal sections of mouse brain fixed tissue and stained with cerebrospinal fluid (CSF) containing anti-IgLON5 antibodies from a patient. **(A–C)** Incubation of IgLON5-knockout (KO) mouse brain tissue with antibodies against IgLON5 shows no reactivity against IgLON5, demonstrating that KO mice do not express detectable IgLON5 protein. **(B)** Magnification of the hippocampus of IgLON5-KO stained with anti-IgLON5 antibodies. **(C)** Magnification of the cerebellum showing no reactivity against IgLON5. **(D–F)** Brain tissue from IgLON5-HET mice showed weaker reactivity than wild-type (WT), as expected, when stained with CSF containing IgLON5 antibodies but reproduced the typical diffuse staining of the neuropil produced by IgLON5 antibodies shown in WT mice **(G–I)**. **(G)** WT mice stained with IgLON5 antibodies depicting the typical pattern of staining of IgLON5 antibodies. **(H)** Magnification of the staining of IgLON5 antibodies in WT mouse hippocampus. **(I)** Magnification of staining pattern of anti-IgLON5 antibodies in WT mouse cerebellum. Immunohistochemistry was counterstained with hematoxylin. Scale bars: mice hippocampus **(G)** 2 mm, **(H)** 250 μm, and **(I)** 250 μm.

**Figure 2:**
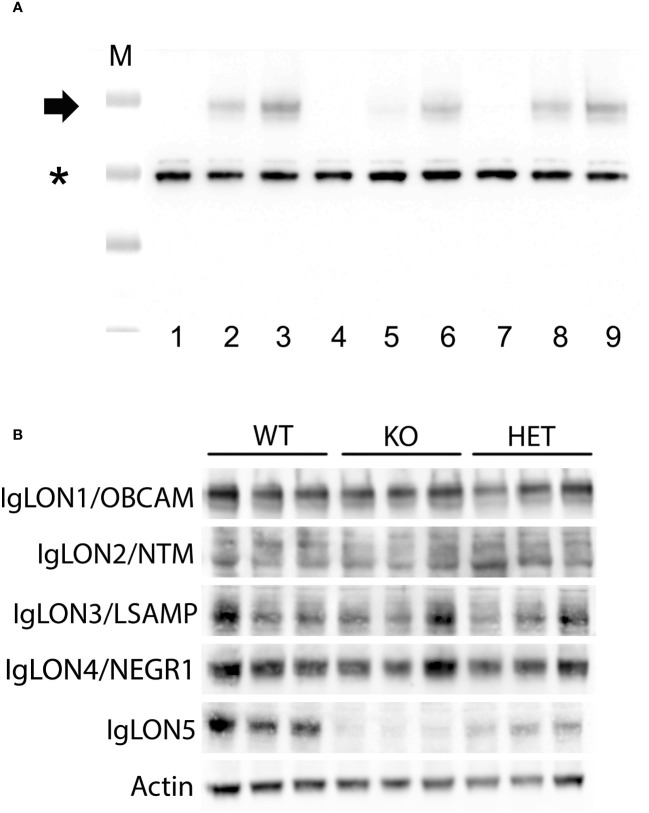
**(A)** Western blotting of lysates from tissue of different mouse brain areas showing the absence of IgLON5 in the IgLON5 knockout mice; cerebellum (lanes 1–3), hippocampus (lanes 4–6), and brainstem (lanes 7–9). Lysates from IgLON5 knockout mice correspond to lanes 1, 4, and 7; lysates from IgLON5-HET mice correspond to lanes 2, 5, 8; lysates from wild-type (WT) mice correspond to lanes 3, 6, and 9. M indicates the molecular weight marker, * indicates band of beta-actin, and the arrow indicates the glycosylated IgLON5 molecular weight. **(B)** Western blotting analysis of mouse cerebellum lysates showing that there is no compensatory effect of the other members of the IgLON family in the absence of IgLON5. Three different mice of each genotype (WT, knockout (KO), and HET) are shown for every antibody incubated (rows, antibodies against IgLON1 to IgLON5 proteins).

IgLON5 protein was undetectable by immunohistochemistry on IgLON5-KO brain sections (**Figures 1A–C**) when incubated with IgLON5-abs from patients (sera or CSF) or with IgLON5 commercial antibody. IgLON5-HET showed weaker reactivity than WT mice by immunohistochemistry (**Figures 1D–F**) but depicts the typical IgLON5 staining pattern identical to that of WT mice (**Figures 1G–I**). IgLON5 was also not detected by Western blotting in tissue lysates obtained from the brainstem, cerebellum, or hippocampus of the IgLON5-KO mice (**Figure 2A**). The other IgLON family members were not upregulated in IgLON5-KO mouse brains (**Figure 2B**). In terms of expression of synaptic receptors, NMDAR, GABABR, or binding partners previously described (CACN2D2A, IGSF21, and KIDINS220) (20) were not altered in IgLON5-KO mice either (data not shown).

**Figure 3:**
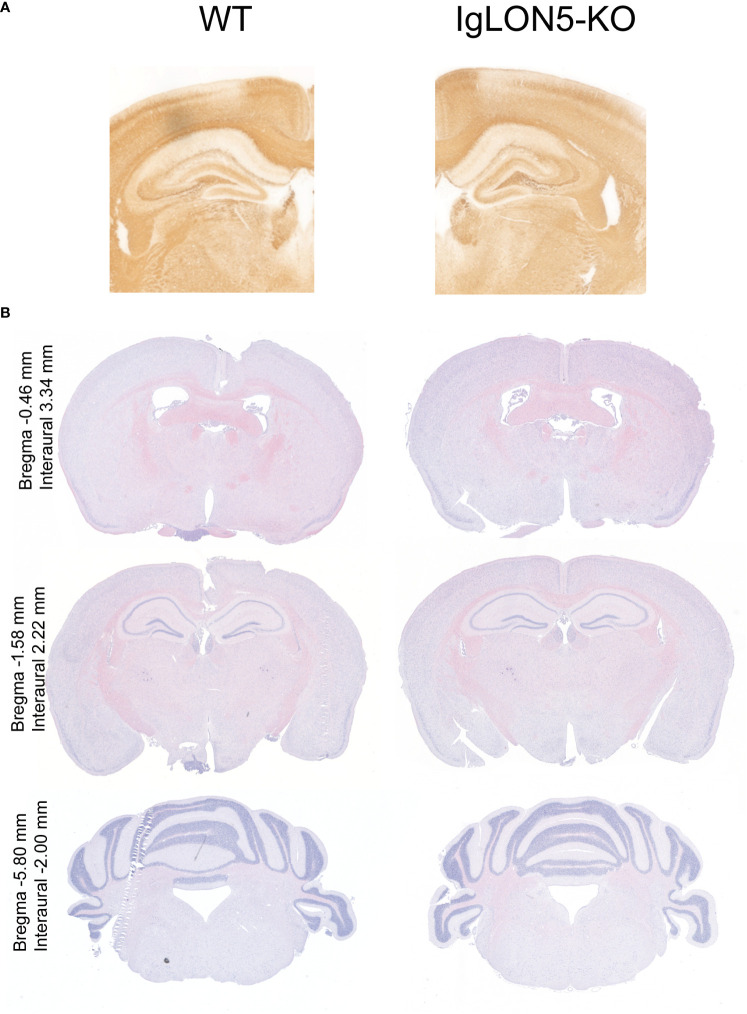
Brain morphology showed no major alterations of IgLON5-knockout (KO) compared to age-matched wild-type (WT) mice (22-month-old). **(A)** Neurofilament immunostaining showed no alteration in the gross cytoarchitecture of the major fiber tracks. **(B)** Histological staining with hematoxylin and eosin of 22-month-old mouse IgLON5-KO tissue compared to age-matched WT mouse tissue also showed no abnormalities in coronal sections of comparable paraffin-embedded brain areas.

**Figure 4:**
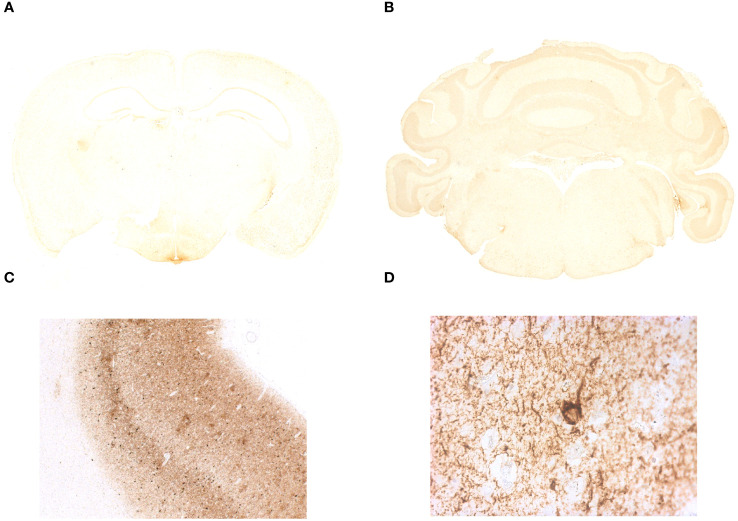
Immunohistochemistry on coronal sections of paraffin-embedded mouse brain tissue did not show the presence of abnormal tau phosphorylation in IgLON5-knockout (KO) mice. **(A, B)** Brain tissue IgLON5-KO mice immunostained with AT8 antibody shows no specific binding to neuropil threads or abnormal tau deposition described in anti-IgLON5 tauopathy. Hippocampus, tegmentum of the brainstem, and hypothalamus show no evidence of pathological changes in IgLON5-KO mice. **(C)** Immunostaining of the prefrontal cortex of a patient with Alzheimer’s disease was run in parallel and serves as positive control. **(D)** Magnification of a positive area for AT8 antibody staining of the Alzheimer’s disease patient tissue showing a neuron with tau aggregation in neurofibrillary tangles.

IgLON5-KO mice were viable and fertile and did not exhibit any evident body morphological alterations. Offspring followed Mendelian sex segregation (100 births). There were no differences in weight between IgLON5-KO and WT animals (p = 0.6927) or in the brain weight at sacrifice (p = 0.1658) in either young animals or those aged 22 months (**Table 1**). We observed no brain abnormalities by conventional hematoxylin and eosin or neurofilament staining of the fiber tracts. Similarly, the area of the ventricles was also similar in IgLON5-KO and WT mice (**Figure 3**). We investigated the presence of phospho-tau deposits in nine 22-month-old IgLON5-KO and nine WT mice (five females and four males). We did not observe tau deposits in IgLON5-deficient animals or controls (**Figure 4**).

### 3.2 IgLON5-KO mice have motor and balance deficits

**Figure 5:**
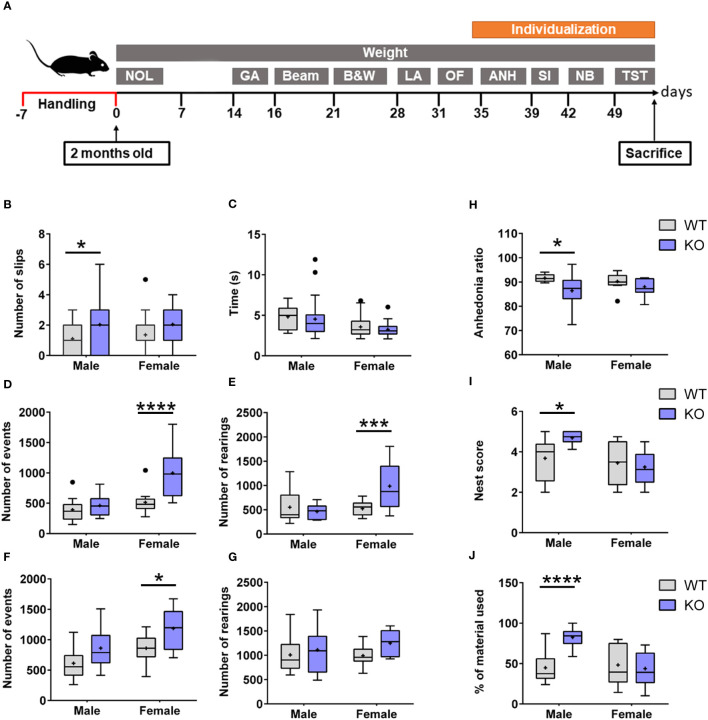
**(A)** Experimental design of the behavioral testing in 2-month-old IgLON5-KO and WT mice to assess the following: 1) Hippocampal-dependent spatial memory: novel object location (NOL). 2) Balance and locomotion: gait analysis (GA), beam balance test (Beam), and locomotor activity (LA). 3) Anxiety: black and white (B&W) and open field (OF). 4) Depressive-like behavior: anhedonia (ANH) and tail suspension test (TST). 5) Social interaction (SI). 6) Nest building (NB). To perform social depressive-like behaviors, nest-building animals were individualized. **(B)** Graphical representation of the quantification of slips in beam balance test separated by sexes and genotypes. IgLON5-KO males exhibited significantly more slips (purple) compared with male WT mice (gray) (*p = 0.03). Although females showed the same trend, they did not reach statistical significance (p = 0.08). **(C)** Time spent to cross the beam was not different between sexes or genotypes. **(D, E)** Spontaneous locomotor activity during daytime measured in activity boxes evidenced hyper-locomotion activity in KO females with an increase in the number of events (genotype, p = 0.0003; *post-hoc* test females, ****p < 0.0001; males, not significant) **(D)** and rearings (genotype, p = 0.026; *post-hoc* test females, ***p = 0.0006) **(E)**. This behavior was not observed in males. **(F, G)** Locomotor activity during nighttime showed similar results to those observed during daytime in IgLON5-KO females with more movement events (genotype, p = 0.0014; *post-hoc* test females, *p = 0.02) **(F)**, and rearing measurements were not significant **(G)**. **(H)** IgLON5-KO males showed less sucrose preference, which is indicative of depressive-like behavior (genotype, p = 0.01; *post-hoc* test males, *p = 0.0487). **(I, J)** Nest-building analyses revealed that IgLON5-KO males built more elaborate nests (genotype, p = 0.01; *post-hoc* test males, *p = 0.0115) **(I)** and used more material (genotype, p = 0.005; *post-hoc* males, **** p < 0.0001) **(J)**. WT, wild type; KO, knockout.

An extensive battery of tests was applied to investigate behavioral deficits in 2-month-old IgLON5-KO in comparison with WT mice (**Figure 5A**). IgLON5-KO mice showed a greater number of slips in the beam balance test, which assesses fine motor coordination, balance, and vestibulomotor function (genotype: F(1, 98) = 8.867; p = 0.0037). IgLON5-KO males lost their balance significantly more times than WT mice (p = 0.03). Although IgLON5-KO females displayed also more slips than WT females, statistical significance was not reached in the *post-hoc* test (p = 0.08) (**Figure 5B**). Time spent to cross the beam was not different between genotypes (KO-WT), indicating that the observed loss of balance cannot be attributed to deficits in muscle strength (**Figure 5C**).

During the daytime, when mice are expected to rest because they are nocturnal, IgLON5-KO female mice exhibited hyperactive behavior. This was evidenced by a consistent increase of back-and-forth movements across the box compared with WT females (p < 0.0001) (**Figure 5D**). The increase in back-and-forth movements of IgLON5-KO females was accompanied by a higher number of daytime rearing (p = 0.0006) (**Figure 5E**). Males did not show statistically significant differences in this test during daytime (**Figures 5D, E**).

During the nighttime, when mice are expected to be more active, IgLON5-KO females crossed the sensor of the activity box more times compared to female WT (p = 0.02), maintaining the hyperactivity condition seen during daytime. IgLON5-KO males showed the same tendency to hyperactivity as females, and more back-and-forth movements were observed during the night compared with WT mice (p = 0.03) (**Figure 5F**). In this case, the number of rearing was not significantly different between males and females (**Figure 5G**).

### 3.3 Male IgLON5-KO mice presented depressive-like and excessive nesting behaviors

Male IgLON5-KO mice manifested lower sucrose preference compared to WT mice (p = 0.0487) (**Figure 5H**). In addition to this depressive-like behavior, male KO mice showed excessive nest-building activity. They built significantly better and larger nests (p = 0.0115) (**Figure 5I**) and used more material compared to WT male littermates (p ≤ 0.0001) (**Figure 5J**). We did not observe differences in the behavior of females.

Overall, these results indicate that IgLON5-KO mice have subtle deficits in fine motor coordination hyper-locomotion activity and depressive-like behavior and that these deficits were sex-dependent. Whereas male IgLON5-KO mice showed more problems with motor coordination and depressive-like behavior, females showed hyper-locomotion activity.

## 4 Discussion

We present the initial characterization of the phenotype and behavior of an IgLON5-KO mouse model.

IgLON5-KO mice showed only subtle abnormalities in the behavioral studies, and results differed between males and females. First, IgLON5-KO males had an increase in slips in the beam balance test, which assesses motor and vestibular function by quantifying the ability to balance on a narrow wooden beam. Although statistical analyses did not reach significance in the female IgLON5-KOs, they showed the same trend as in males. Our results suggest that IgLON5 has a physiological role in fine motor coordination and balance; these findings support the possibility that IgLON5 autoantibodies can interfere with IgLON5’s function and may be pathogenically involved in the gait instability frequently noted in this disorder. This is also consistent with the results of a recent study of passive antibody transfer where intracerebral injections of IgLON5-IgG from patients into the nigrostriatal dopaminergic pathway of mice produced sustained motor impairment (22). Behavioral studies reported in KOs of other IgLON family members did not describe deficits in balance or motor coordination (27–30) (**Table 2**).

**Table 2:** Comparison of the behavior of IgLON5-KO with five studies reported on NMT-KO, LSAMP-KO, and NEGR1-KO.

	IgLON5-KO female	IgLON5-KO male	NTM-KO (30)	LSAMP-KO (27, 28)	NEGR1-KO (23, 31)
Behavior					
**Anxiety**				* ↓	↑^¶^
**Depressive-like behavior**		↑	ND	ND	↑^¶^
**Social interaction**				↓	↓
**Motor activity**	↑	↑^&^		*↑	
**Memory/learning**			↓ **	↓^$^	↓
**Motor coordination**		↓	ND		

Green cells indicate no differences between KO and WT mice and red cells indicate some disparity in results.

WT, wild type; KO, knockout.

^&^Increased motor activity observed only at night.

^*^In response to novel environments.

^**^Mild deficits in cognitive challenging emotional learning tasks.

^$^Impaired spatial memory in one study (Qiu, 2010) (28).

^¶^Abnormal (Noh, 2019) (31). ↑, increased. ↓, decreased. ND, not determined.

Second, female IgLON5-KOs showed hyperactivity during darkness and also during the light phase when mice are supposed to be resting. Deletion of the other IgLONs in mouse models also showed hyperactivity, especially in stressful conditions (23, 27) (**Table 2**). This phenotypic feature together with a depressive-like behavior and an obsessive nest-building activity observed in IgLON5-KO males could indicate a role of IgLON5 deficiency in psychiatric-related symptoms (32).

Our results on the behavior of IgLON5-KO mice are similar to those reported in other IgLON-KO mouse models, which also showed subtle behavioral abnormalities, related to social behavior, learning, and locomotor activity (**Table 2**). These behavioral alterations have suggested that IgLONs are implicated in the pathogenesis of psychiatric disorders. However, psychiatric symptoms are not a major complaint in anti-IgLON5 disease. In a series of 53 patients with anti-IgLON5 disease, only nine (17%) developed psychosis or hallucinations (33). However, the two IgLON family members, Negr1 (IgLON4) and LSAMP (IgLON3), which showed more behavioral alterations in the KO mouse model, have been implicated in several psychiatric disorders (23, 27, 34). Recent meta-analyses using the Genome-Wide Association Study (GWAS) have identified a single-nucleotide polymorphism (SNP) of Negr1 gene associated with the diagnosis of major depression (35). Also, Negr1 has been proposed as a biomarker of major depression because CSF Negr1 levels were elevated in patients with this disorder (36). Similarly, SNPs in LSAMP gene have been associated with major depression and panic disorders (37). In contrast, NTM (IgLON2) KO mice only showed minor emotional-related learning deficiencies in the active avoidance test, and in human genetic studies, NTM has not been identified as a risk for psychiatric diseases (30).

IgLON5-KO mice were viable and fertile and did not show any appreciable morphologic or cerebral alterations. Gross and microscopic neuroanatomy examination of IgLON5-KO and WT mice did not reveal differences regarding principal fiber tracks or volume of the ventricles, in either young adult or older mice. Studies of KOs of other IgLONs reported also no major abnormalities except for Negr1, which showed substantial volumetric alterations in the ventricular space (23). Although ventriculomegaly was observed in a patient with anti-IgLON5 disease (38), the brain MRI of most (>90%) patients showed a similar degree of atrophy as that of age-matched controls or only a mild atrophy of the brainstem (38, 39).

Finally, we investigated whether aged IgLON5-KO mice developed signs of neurodegeneration. In these mice, the deficiency in IgLON5 did not lead to distinct pathological signs such as neuronal loss or tau hyperphosphorylation, which have been reported in the autopsies of some patients (1, 7). This observation suggests that other factors in addition to IgLON5 loss, for example, inflammatory changes associated with IgLON5 autoimmunity, may be important in the development of the neurodegenerative alterations observed in some patients. Therefore, it is possible that the antibody effects observed *in vitro* are necessary to cause the disease; thus, IgG1-dependent cross-linking and internalization of IgLON5 clusters would be followed by alteration of the cytoskeletal architecture and would lead ultimately to tau aggregation and phosphorylation through common pathways described for neurodegenerative diseases. The role of IgG4 IgLON5 antibodies in the disease is less clear because *in vitro* studies have shown that alteration of the interactions of the IgLON5 protein with its binding partners, which is the main pathogenic mechanism described for IgG4 antibodies, was not specific to a subclass. However, IgG4 antibodies may be relevant to the pathogenesis of the disease because a recent autopsy study has reported the presence of abundant IgG4 deposits in areas of the brain where IgLON5 is highly expressed and whose dysfunction may explain the clinical symptoms (10). Our study has limitations because we have not conducted a detailed sleep evaluation, and the clinical phenotype of IgLON5 deficiency is not fully coincident with the core of symptoms of the disease; however, these symptoms are also variable among patients. Another limitation is that we focused our studies on the behavior and brain of mice, but IgLON5 is also present in systemic tissues like muscle fibers, which were not investigated and should be explored in future studies.

To summarize, our model of IgLON5-KO shows subtle deficits in fine motor coordination, balance, and behavioral alterations similar to those seen in KOs of other IgLON family members. These changes were in part sex-dependent. However, the IgLON5-KO mouse model does not reproduce the clinical phenotype or the tauopathy reported in anti-IgLON5 disease.

## Data availability statement

The original contributions presented in the study are included in the article/supplementary material. Further inquiries can be directed to the corresponding author.

## Ethics statement

The animal study was approved by Comitè Ètic d’Experimentació Animal, Universidad de Barcelona. The study was conducted in accordance with the local legislation and institutional requirements.

## Author contributions

JL: Conceptualization, Investigation, Methodology, Writing – review & editing. AS: Investigation, Methodology, Writing – review & editing. MA: Methodology, Writing – review & editing, Investigation. EM: Investigation, Methodology, Writing – review & editing. LM-P: Conceptualization, Methodology, Writing – review & editing, Investigation. AG-S: Investigation, Methodology, Writing – review & editing. FM: Methodology, Writing – review & editing, Investigation. JD: Writing – review & editing, Investigation, Methodology. FG: Investigation, Supervision, Writing – review & editing, Methodology. LS: Conceptualization, Funding acquisition, Investigation, Methodology, Supervision, Writing – original draft, Writing – review & editing.
